# Physical literacy among older adults: a scoping review of definition, attributes, contributing factors, consequences and interventions

**DOI:** 10.3389/fpubh.2025.1678171

**Published:** 2025-11-05

**Authors:** Yawei Yu, Yu Duan, Hong Guo, Yiping Chen

**Affiliations:** School of Nursing, Beijing University of Chinese Medicine, Beijing, China

**Keywords:** physical literacy, older, older adults, scoping review, review

## Abstract

**Background:**

Physical literacy (PL) is increasingly recognized as a foundational component of lifelong physical activity participation and healthy aging. However, research on PL in older adults remains scattered and lacks synthesis across domains.

**Objective:**

To systematically map the current research landscape on physical literacy in older adults, including its definitions, core attributes, contributing factors, health-related outcomes, and intervention practices.

**Method:**

Six databases (PubMed, Web of Science, Embase, CINAHL, SPORTDiscus, and PsycINFO) were searched from inception to May 2025. Additional grey literature was identified through Google Scholar and reference screening. Adopted Arksey and O′Malley’s five-stage methodological framework, two reviewers independently screened and extracted data focusing on definitions, attributes, antecedents, consequences, and interventions related to PL in older adults. Findings were synthesized using Braun and Clarke’s descriptive thematic analysis approach.

**Results:**

Eighteen studies were included, which were published between 2019 and 2025, spanning medicine, psychology, and social sciences. The concept of PL among older adults did not have a consolidated definition but was identified through five defining attributes: Physical competence, Motivation and confidence, Knowledge and understanding, Affective and cognitive engagement and Environmental and social interaction. Influencing factors clustered into four categories: biomedical, psychosocial and behavioral habits, sociocultural environment, and education and early experiences. PL was positively associated with physical and mental health, willingness to engage in physical activity, successful aging, and public health outcomes. Intervention strategies included education-based programs, functional exercise training, dance, and exergaming.

**Conclusion:**

Physical literacy is essential for supporting active aging. Standardized assessment tools and integrated interventions are needed to enhance PL among older adults. Future efforts should build upon the attributes of PL among older adults to develop or refine multidimensional assessment tools specifically designed for older adults, ensuring their cultural relevance and practical applicability.

## 1 Introduction

The decline in physical function, high prevalence of chronic diseases, and widespread sedentary behavior among older adults have positioned physical activity promotion as central to improving their quality of life. Although the benefits of physical activity are widely acknowledged, global participation rates among older adults remain low (1). Within this context, physical literacy (PL) has emerged as a critical concept for enhancing motivation toward physical activity (2), encompassing the physical, psychological, cognitive, and social capacities necessary for engaging in movement. PL is a holistic and developmental concept, emphasizing not only the ability to move competently but also the individual’s understanding of movement significance, self-efficacy, and interaction with the sociocultural environment.

The concept of PL is informed by philosophical theories, including monism, existentialism, and phenomenology. Based on different philosophical theories, the concept of PL is various. From the beginning of the century, a growing use of and consensus around PL has been evidenced, with Whitehead and IPLA’s definition being the major source of influence (3). Many researchers adopting the “Whiteheadian” approach, considering PL as a multidimensional construct containing the motivation, confidence, physical competence, knowledge and understanding to maintain physical activity throughout the life course.

PL has different characteristics in each age stage. As a core concept for promoting physical activity, PL has gradually expanded in its application from youth and adults to the older adult population. However, current research investigating physical literacy has almost exclusively involved children and adolescents, only relatively little evidence of PL being used to support older adults in achieving the PA guidelines, which may ignore the importance of older adults. Particularly for older adults, the development of PL is influenced by multiple factors such as physical health status, early-life experiences, social support, and educational background (4). Compared to younger individuals, older adults face distinct needs and challenges in the development of physical literacy. Physiologically, older adults experience natural declines in strength, balance, coordination, and flexibility, making the maintenance or recovery of basic daily functions (e.g., walking, standing, stair climbing) particularly crucial (5). Cognitively, age-related slowing of information processing or mild cognitive impairment may impair older adults’ ability to acquire new knowledge or adapt to physical activities, making self-awareness of bodily changes and aging processes integral components of PL (6). Emotionally and socially, significant life transitions such as retirement, loss of intimate relationships, and altered social roles profoundly impact older adults’ motivation, self-identity, and willingness to participate in physical activities (7).

With the often-discussed importance of lifelong development, researchers are progressively developing PL frameworks tailored specifically for older adulthood, emphasizing functional movements, adaptive behaviors, emotional support, and social engagement. This theoretical expansion illustrates the intrinsic consistency of PL as a health-promotion philosophy across the life course, serving not only the skill development of youth but also providing structured support for healthy aging (8). Despite the attention to PL in older adults, there is a dearth of research that has explored PL in older adults. Due to the diversity of the concept of PL itself, different studies have different understanding on PL in older adults, resulting in the concept of PL in older adults is still unclear. The lack of clarity undermines its operationalization for older adults. Developing public health strategies, policies and guidelines, as well as intervention programs, requires a clear understanding of what components constitute physical literacy, and how it can be observed and assessed.

To address these gaps, this study systematically maps the existing literature on physical literacy in older adults, with particular attention to its characteristics, attributes, contributing factors, consequences, and intervention strategies. Through this comprehensive synthesis, we aim to provide theoretical insights to inform future research, guide clinical nursing practice, and support policy development for healthy aging.

## 2 Methods

This review adopted Arksey and O’Malley’s (9) five-stage methodological framework to explore definitions, attributes, influencing factors, consequences, and interventions associated with PL in older adults. This framework is useful in clarifying complex concepts and has been widely used in emergent fields of health research where available evidence is heterogeneous. The review process adhered to the Preferred Reporting System for Meta-Analysis for Scoping reviews (PRISMA-ScR) (10).

### 2.1 Identifying research questions

The research team collaboratively formulated the following research questions: (1) What are the definitions and core attributes of physical literacy in older adults? (2) What factors influence physical literacy in older adults? (3) What consequences are associated with physical literacy for older adults? (4) What interventions related to physical literacy currently exist for older adults?

### 2.2 Identifying relevant studies

Two researchers systematically searched six databases (PubMed, Web of Science, Embase, CINAHL, SPORTDiscus, and PsycINFO) using combinations of the keywords “aged/geriatrics/older/elderly/senior” and “physical literacy,” covering publications from database inception to May 2025. To minimize publication bias and comprehensively collect evidence, additional manual searches, including grey literature, were conducted using Google Scholar. Search strategies were adapted for each database (see Appendix 1: Search Strategy Table). Reference lists of all included studies were screened to identify additional relevant articles that might have been overlooked during the database searches.

### 2.3 Inclusion and exclusion criteria

Articles were included if they met the following criteria: (1) study addressed the concept, theoretical framework, associated factors, or interventions related to physical literacy; (2) study population aged 60 years or older, or or had an average age of 60 or older; (3) study conducted in diverse settings or populations; (4) study published in peer-reviewed journals using any study design; and (5) written in English.

Studies in which physical literacy was not the primary focus were excluded.

### 2.4 Study selection

Initially, two researchers independently screened article titles and abstracts against the inclusion and exclusion criteria. Potentially relevant articles proceeded to full-text review, where the same selection criteria were applied. Disagreements were resolved through discussion with a third researcher.

### 2.5 Charting the data

Data were extracted from included studies, focusing on author/year, study design, antecedents, attributes, consequences, and intervention strategies related to older adults’ physical literacy (see Table 1 for details).

**Table 1:** Description of articles included in the scoping review (*n* = 18).

Author, year	Type of article	Attribute	Contributing factors	Consequence	Intervention
Huang et al. (2022) (12)	a cross-sectional study		PB; EE	(1)	
Jones et al. (2018) (8)	an iterative and mixed-methods consensus development process, Delphi survey	MC; PC; KU	SE		
Kim Wai Sum et al. (2024) (13)	a cluster randomized controlled trial	PC; MC; ESI; KU		(3)	
Lloyd et al. (2024) (14)	a position paper	PC; MC; ESI; KU	PB; SE		
Magrath et al. (2024) (15)	an observational study, Ethnographic fieldwork practices		PB; EE		★
Matsunaga et al. (2025) (16)	an cross-sectional survey	PC; MC; ESI; KU			
Naylor et al. (2024) (17)	an cross-sectional survey	PC; MC; ESI; KU			
Naylor et al. (2025) (26)	a two-phase cross-sectional study		SE	(2)	
Petrusevski et al. (2022) (4)	a systematic integrative review	PC; MC; ESI; KU	SE; PB; EE	(3)	★
Roetert et al. (2019) (2)	a review	PC; MC; ESI; KU	BM	(3)	★
Wang et al. (2023) (18)	a mixed methods research design	PC; MC; ESI; KU	PB	(2)	
Zhang et al. (2022) (19)	Modified Delphi Study	KU; PC			
Bopp et al. (2022) (20)	A Systematic Review	PC; KU; MC			★
Campelo et al. (2022) (21)	a mixed methods research design	PC; MC; ESI; KU	SE; BM		★
Liu et al. (2025) (22)	an cross-sectional survey		BM; PB	(2)	
Young et al. (2019) (23)	a concept analysis	PC; MC; ESI; KU	SE; PB	(1); (3)	
Paglione et al. (2024) (24)	a qualitative research				★
Sæther et al. (2024) (25)	a qualitative research	KU; ESI			

Contributing factors: BM (Biomedical factors), PB (Psychological and behavioral habits), SE (Social environmental factors), EE (Educational and early experiences). Attributes: PC (Physical competence), MC (Motivation and confidence), KU (Knowledge and understanding), ACE (Affective and cognitive engagement), ESI (Environmental and social interaction). Consequences: (1) Improved physical and mental health, (2) Enhanced physical activity participation. (3) Successful aging and public health benefits. ★: The article mentioned interventions about PL.

### 2.6 Collating, summarizing, and reporting the results

Data were analyzed using Braun and Clarke’s (11) descriptive thematic analysis approach. First, researchers establish a deep connection with the data through careful reading the articles, highlighting key concepts related to PL in older adults, with a focus on attributes, contributing factors, consequences, and interventions. Next, open coding is performed, where meaningful segments of data are labelled as initial codes. After generating initial codes, researchers organise them into potential themes through induction and aggregation. The researchers looked through topics before defining and naming them, theme names should be engaging and succinct, conveying their core meaning accurately. Finally, a comprehensive summary and synthesis of findings related to PL in older adults were provided. To address confirmability, multiple authors participated in the data analysis process, promoting objectivity in theme development. Regular discussions among the research team minimised the impact of individual researcher bias. Throughout this process, researchers maintain reflexivity, paying attention to their theoretical stance and analytical trajectory to ensure that the analysis is both in-depth and logically transparent.

## 3 Results

### 3.1 Characteristics of included studies

**Figure 1:**
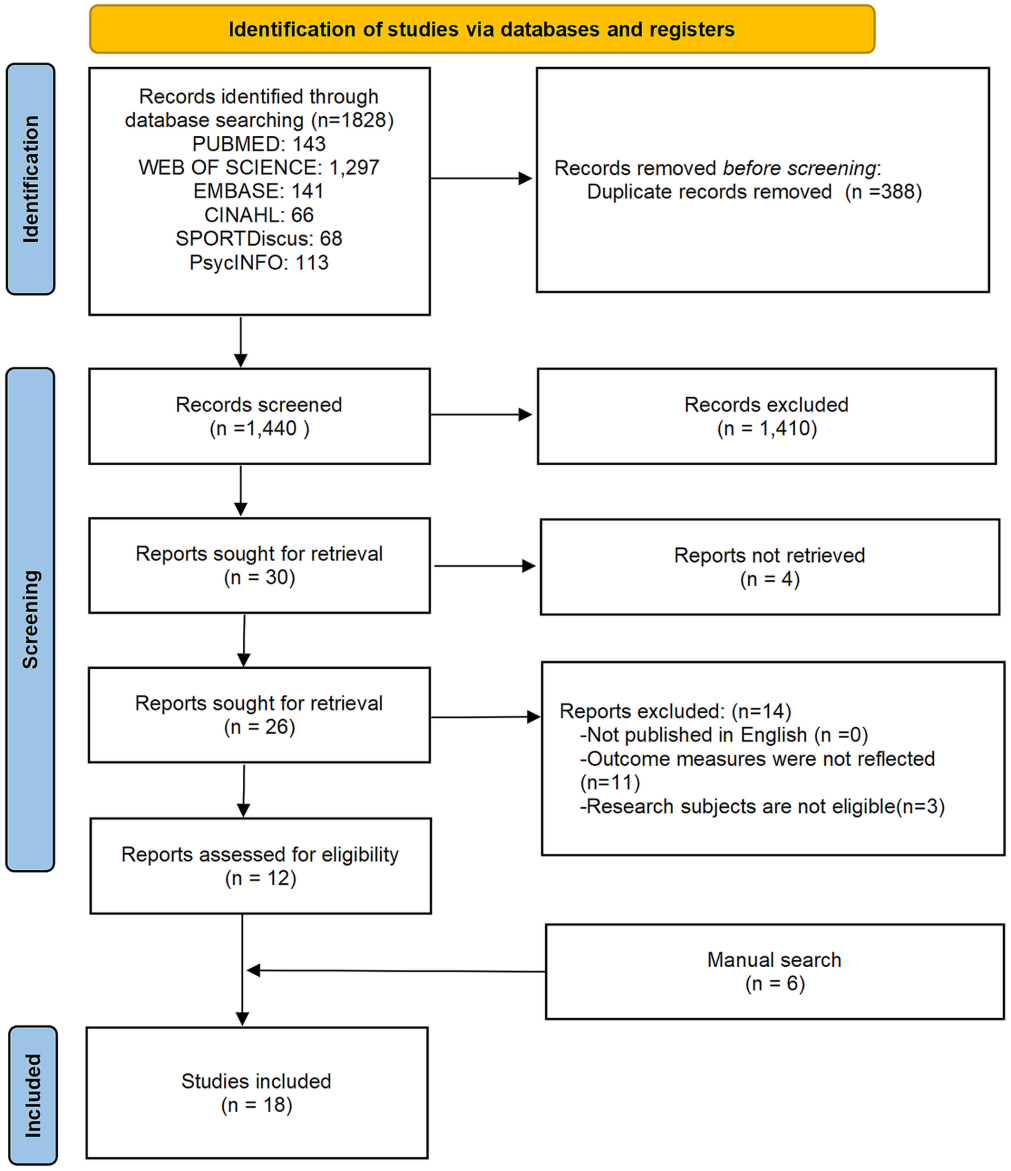
PRISMA diagram of the literature search.

Of the 1,440 articles remaining after duplicates removal, 1,410 were excluded due to irrelevant titles or abstracts. Full texts of the remaining 30 articles were independently reviewed by two researchers. Among these, four full texts were unavailable, 11 did not focus on the concept, theoretical framework, influencing factors, or interventions related to physical literacy, and three studies had inappropriate study populations. Additionally, six studies were identified through manual searching. Finally, 18 studies were included, covering diverse fields such as medicine, psychology, and social sciences. The detailed selection process is shown in Figure 1 (PRISMA flowchart).

**Figure 2:**
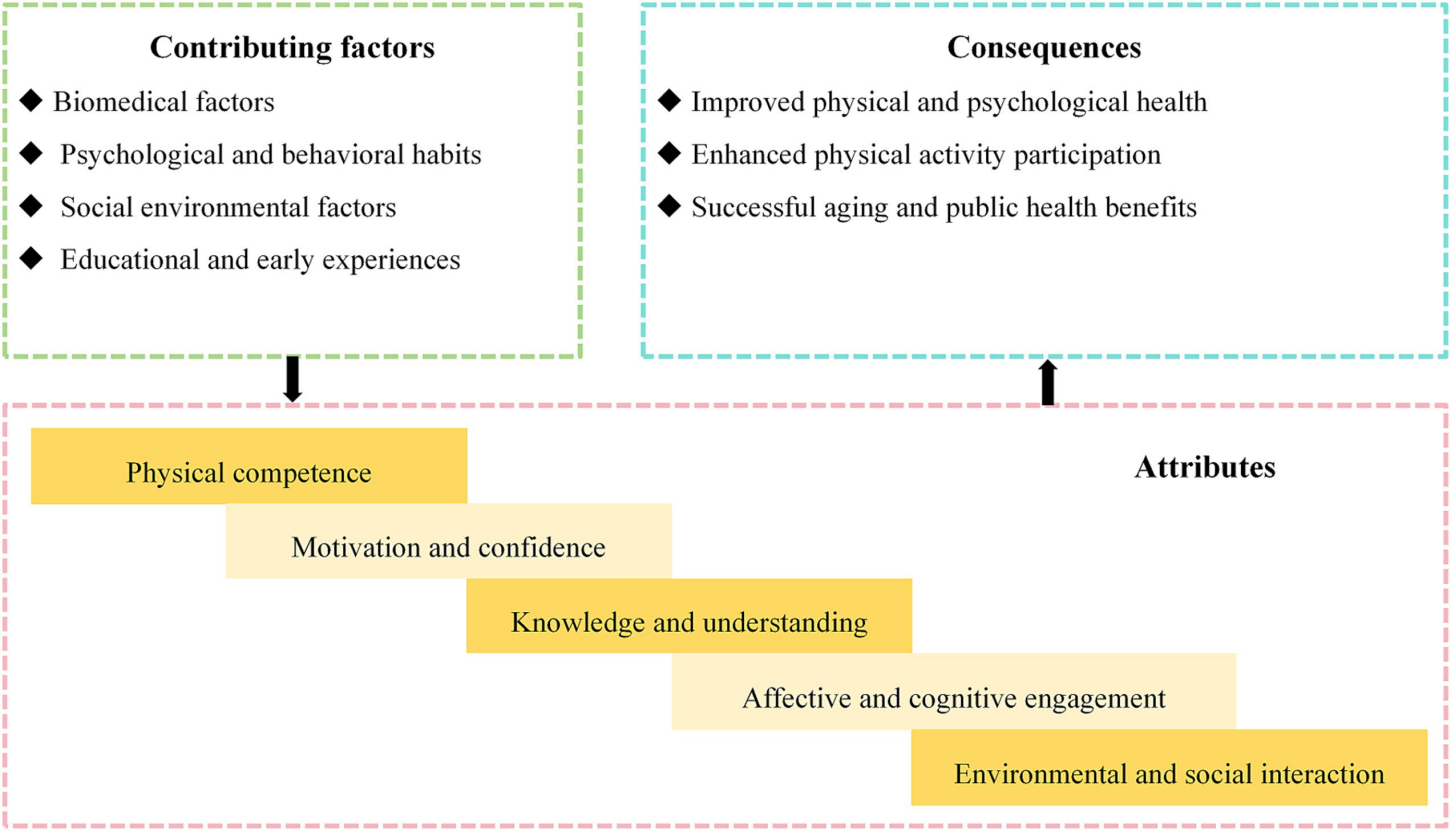
Thematic analysis results of included studies.

Included articles were published between 2019 and 2025, encompassing reviews (*n* = 5), qualitative studies (*n* = 4), cross-sectional studies (*n* = 5), mixed-method studies (*n* = 2), interventional studies (*n* = 2). Studies originated from the USA (*n* = 2), Canada (*n* = 6), Australia (*n* = 3), China (*n* = 5), Japan (*n* = 1), and Norway (*n* = 1). Thematic analysis results including attributes, influencing factors, consequences, and interventions related to physical literacy in older adults are presented in Table 1 and Figure 2.

### 3.2 Definitions of physical literacy in older adults

The concept of PL among older adults did not have a consolidated definition but was identified through five defining attributes: Physical competence, Motivation and confidence, Knowledge and understanding, Affective and cognitive engagement and Environmental and social interaction. In the included articles, most definitions of PL in older adults were dominated by Whitehead framework or the definition provided by the International Physical Literacy Association (IPLA). These articles emphasized motivation, confidence, physical competence and knowledge/understanding, considering PL as a developmental concept through the whole lifespan. Seventeen articles (2, 4, 8, 12–25) referred to Margaret Whitehead’s definition, with four articles (8, 12, 16, 18) explicitly adopting and expanding upon it (8, 16, 18). As a pioneer, Whitehead consistently advocates refining the concept of physical literacy (13), recently aligning it with the definition provided by the International Physical Literacy Association (IPLA): “the motivation, confidence, physical competence, knowledge and understanding to value and take responsibility for engagement in physical activities for life” (4, 16, 17, 20, 21, 23). What’s more, some studies (2, 4, 8, 13, 14, 16, 17) extended the definition of PL to contexts and environments, linking individual PL to external dimensions such as interpersonal, organizational, community and policy to illustrate how older persons stayed active in specific environments. Currently, the core attributes of PL among older adults in existing articles were relatively consistent, but there remained a lack of unique and specific “adaptive adjustments” to the older adults. Discussions about physical function changes, multiple comorbidities, and strategies for modifying physical activity in diverse environments just remained at the conceptual level, lacking detailed operational specifications and unified terminology.

### 3.3 Attributes of physical literacy in older adults

Physical literacy in older adults is a multidimensional concept consisting of five key attributes: physical competence, motivation and confidence, knowledge and understanding, affective and cognitive engagement, and environmental and social interaction.

#### 3.3.1 Physical competence

Physical competence forms the foundation of older adults’ physical literacy. Eleven articles (4, 8, 13, 16–21, 23) identified physical competence as the fundamental movement skills necessary for engaging in physical activities (8, 20), including balance, coordination, strength, flexibility, and agility.

#### 3.3.2 Motivation and confidence

Eight articles (4, 8, 14, 16–18, 21, 23) highlighted motivation and confidence. From a PL perspective, motivation closely relates to confidence. Older adults select activities aligning with personal interests, abilities, and age-related capacities, enhancing confidence in physical activities (2). Higher exercise-related self-efficacy strongly determines older adults’ persistence in physical activities (8). Though similar to younger adults in relying on past experiences, older adults’ motivation and confidence differ due to health status and expected health benefits, making it crucial to understand their past activity experiences and health expectations (8).

#### 3.3.3 Knowledge and understanding

Knowledge and understanding involve grasping the health-promoting effects of physical activities and understanding safe, effective, age-appropriate methods of exercise. Nine studies (2, 4, 8, 16–20, 23) discussed related content, focusing on age-related physical changes (4, 16), benefits for healthy aging (8), and proper exercise methods (8). Limited knowledge of recommended activity levels and benefits often hinders older adults’ participation (2, 8, 19). Mere repetitive movements without understanding fail to sustain enjoyment and consistent participation (19).

#### 3.3.4 Affective and cognitive engagement

Seven studies (4, 14, 16, 18–20, 23) addressed affective and cognitive engagement, emphasizing emotional investment and cognitive involvement, including enjoyment, self-regulation, and awareness of physical conditions. Building on activity-related knowledge (19), maintaining motivation, enjoyment, and self-efficacy fosters positive physical activity behaviors (16). Selecting enjoyable activities enhances older adults’ activity experiences, creating positive behavioral cycles (2, 19).

#### 3.3.5 Environmental and social interaction

Eight studies addressed social interaction (4, 14, 16, 17) and environmental perception (2, 4, 21). Physical literacy is inherently situational and social, influenced by sociocultural norms, expectations, and physical and natural environments (8). Older adults with PL can effectively navigate environments (e.g., stairs, uneven surfaces) and engage in social contexts. Positive social interactions significantly encourage activity participation, increasing motivation among peers (2, 17).

### 3.4 Contributing factors of physical literacy in older adults

Four contributing factors were identified: biomedical, psychological and behavioral habits, social environment, and educational and early-life experiences.

#### 3.4.1 Biomedical factors

Two studies mentioned biomedical factors. Biomedical factors, including current health status and physical ability, significantly impact older adults’ PL. Aging, characterized by declining physical and cognitive functions, affects activities of daily living and increases risks such as falls (21). Age-related functional declines decrease older adults’ physical competencies, affecting environmental perception capabilities. Liu et al. (22) found older women performed worse in Senior Functional Fitness Tests with increasing age, indicating reduced limb functionality.

#### 3.4.2 Psychological and behavioral habits

There were four studies mentioned psychological and behavioral habits. These factors encompass attitudes towards physical activities, perceived physical well-being, and daily habits and preferences. Positive attitudes sustain lifelong engagement (14). Conversely, frustrations or perceived failures negatively impact participation (21). Huang et al. (12) reported weak correlations between perceived physical literacy and physical well-being. Adults choosing diverse, enjoyable activities demonstrate higher PL, sustained by routine exercise and positive attitudes toward physical activity (4, 18, 22).

#### 3.4.3 Social environmental factors

These include social support and community/policy environments. Six studies (8, 14, 21, 23, 26) emphasized the importance of extensive, supportive interpersonal relationships. Community environments must facilitate activities through infrastructure, accessible opportunities, diverse programs, and supportive, culturally relevant policies (4, 8, 26).

#### 3.4.4 Educational and early experiences

Two studies mentioned educational and early experiences. Physical literacy is influenced by educational attainment (12) and childhood activity experiences (4). Higher educational levels correlate positively with better PL knowledge and physical competence (12). Early childhood PL development shapes lifelong activity habits and skills (4).

### 3.5 Consequences of physical literacy in older adults

Three consequences were identified: improved physical and psychological health, enhanced activity participation, and successful aging and public health benefits. Two studies mentioned the consequence of improving physical and mental health through regular activity (12, 23). Three studies mentioned the consequence of enhancing motivation, behavior, and consistent engagement in physical activities (15, 23, 26). Four studies mentioned the consequence of promoting successful aging and broader public health improvements (2, 4, 13, 23).

### 3.6 Interventions

Six studies (2, 4, 15, 20, 21, 24) discussed interventions, some of them (2, 20, 24) emphasized the importance of professional educators/coaches in fostering PL. These studies contained three intervention types: community dance programs, functional resistance training, and technology-assisted interventions. Community dance served as a key platform for PL development among older adults. Dance interventions were identified for improving communication and PL (24), offering multidimensional engagement for older adults through artistic exercise that balances fun with functional benefits. One study recommended functional resistance training (2), by guiding the older adults to bridge, squat and heel raise, the physical ability and daily activity autonomy of the older adults could be gradually improved. Another article demonstrated that exergaming (Active Video Games, AVG) significantly improved older adults’ functional ability (Timed Up and Go test) and health-related motivation (21). Through virtual interaction, the fun of exercise for the older adults were enhanced, the resistance to the monotony of traditional exercise mode could be reduced. These interventions mostly aimed at improving the physical function and activity ability of the older adults, providing a way to participate in social entertainment for older adults. However, the attention to the attitude and preferences of the older adults to participate in physical activities were insufficient, ignoring the psychological and behavioral habits of PL.

## 4 Discussion

This study systematically synthesizes recent research on physical literacy (PL) among older adults, identifying five core attributes and four key antecedent factors, and summarizing related health outcomes and intervention characteristics. Based on this study, PL among older adults is a multidimensional concept that involves attributes related to Physical competence, Motivation and confidence, Knowledge and understanding, Affective and cognitive engagement and Environmental and social interaction. PL among older adults can improve physical and psychological health, enhanced activity participation, and successful aging and public health benefits. Originally emerging from physical education targeting adolescents, PL has increasingly expanded into adulthood and older age groups, evolving into a significant health concept for promoting active aging and enhancing quality of life. Nevertheless, current research on older adults’ physical literacy remains hindered by vague conceptual definitions, a lack of standardized measurement tools, and fragmented intervention approaches. Therefore, it is essential to re-examine the essence of older adults’ PL and explore effective pathways to improve it from a life-course and multidimensional health perspective.

This study highlights five interrelated core attributes of older adults’ physical literacy: physical competence, motivation and confidence, knowledge and understanding, affective and cognitive engagement, and the capacity for environmental and social interaction. Collectively, these attributes form a cognitive-behavioral-social framework transitioning older adults from mere physical “capability” toward sustained participation. Physical competence is foundational, directly influencing older adults’ ability to perform daily physical activities. Numerous studies indicate that declines in strength, balance, and flexibility significantly constrain older adults’ motivation and ability to engage in physical activity, also increasing their risks for falls, frailty, and disability (27). Motivation and confidence act as internal drivers, greatly influencing whether older adults initiate participation in physical activities. Previous research has demonstrated that higher self-efficacy among older adults significantly predicts sustained regular exercise (28). Conversely, fear of falling, exercise anxiety, or negative past experiences tend to undermine their confidence (29).

At the cognitive level, knowledge and understanding reflect whether individuals recognize the importance of physical activity and grasp safe, scientifically sound exercise methods. This study reveals that insufficient health literacy and cultural biases lead some older adults to misconceptions such as “being too old to exercise,” hindering their PL development (30, 31). Affective and cognitive engagement emphasizes emotional experience and psychological immersion during physical activities. Closely related to enjoyment, social interactions, and ritualization, this dimension substantially influences older adults’ sustained participation (32). Furthermore, environmental and social interaction capabilities represent the extent to which older adults can translate their PL into practical behaviors in everyday life (21). Community facilities, family support, transportation convenience, and sociocultural environments profoundly affect the initiation and maintenance of physical activities.

These attributes are not isolated but dynamically interconnected. For example, increased knowledge can enhance confidence, pleasurable experiences can strengthen motivation, and social interactions can sustain emotional engagement and habitual behaviors. Thus, interventions designed to promote PL should adopt a multidimensional collaborative approach rather than focusing exclusively on a single dimension.

Additionally, this review identified four main antecedent factors influencing older adults’ PL: biomedical factors, psychological and behavioral factors, social environmental factors, and educational and early-life experiences. Biomedical factors, such as chronic diseases, fitness levels, pain, and cognitive function, form critical starting points for determining older adults’ PL. Individuals with poorer physical health and functional limitations typically exhibit lower PL. Psychological and behavioral factors also profoundly impact PL, influencing perceptions of physical well-being and shaping long-term daily habits and activity preferences. These factors determine whether older adults view physical activities as meaningful components of their lives and influence their sustained engagement. Social environmental factors, representing external resource systems, significantly shape PL. Family support, neighborhood interactions, community facilities, cultural identity, and gender role expectations all positively or negatively influence older adults’ willingness and opportunities for physical activities (33). Educational background and early-life experiences determine cumulative physical activity levels and cognitive patterns established before older adulthood. Previous studies have shown that childhood and adolescent physical activity experiences significantly affect physical activity preferences and behaviors in adulthood (34).

Regarding health outcomes, this review found PL closely associated with older adults’ physical function, mental health, fall risks, social participation, and quality of life (12, 23, 26). Older adults with higher PL levels not only participate more regularly in physical activities but also experience enhanced physical and emotional benefits from exercise, creating a positive feedback loop (15, 26), which is closely related to the core idea of successful aging. Successful aging has been called “vital aging” or “active aging” or “productive aging” with the implication that later life can be a time of sustained health and vitality where older people contribute to society rather than merely a time of ill health and dependency (35). With the evidence indicating a positive relationship between physical literacy and healthy aging, current studies developed physical literacy interventions based the concept of PL in older adults, aimed at fostering good exercise habits and daily behavioral routines among older adults. PL was further identified as a key mechanism influencing “successful aging” (2, 4, 13), highlighting its significance as a potential intervention target.

In terms of interventions, multiple studies have attempted to enhance older adults’ PL through physical literacy education, dance training, and resistance training. However, most interventions have predominantly targeted physical competence, with limited systematic attention to motivational stimulation, knowledge building, and emotional engagement (2, 4, 15, 20, 21, 24). Additionally, interventions have generally lacked sufficient personalization, failing to adequately consider the diverse needs and barriers faced by older adults with varying PL levels. Future research should aim to develop stratified, modular intervention frameworks that integrate physical competence, motivation building, educational knowledge, and social resources, thereby enhancing the systematic, adaptive, and sustainable nature of interventions.

### 4.1 Limitations and future directions

Despite highlighting the positive significance of physical literacy (PL) for older adults’ health, this review identifies several notable limitations. First, only literature containing specific keywords in the abstract were included in the manual analysis and synthesis. This could have led to the exclusion of relevant literature that did not include those keywords in the abstract, particularly if the abstracts were not well-structured and did not accurately represent the key findings of the study. Future studies could consider broader inclusion criteria and more thorough screening methods to minimize the risk of missing relevant literature.

Second, standardized measurement tools for assessing PL have yet to be established. On the one hand, due to the differences in the definitions of the concept of PL by different philosophical foundations, the focus of the assessment tools of PL for the older adults varies. On the other hand, most existing studies predominantly focus on the dimension of “physical competence,” while cognitive, emotional, and social interaction dimensions receive insufficient attention, neglecting the integrity of PL, making it difficult to comprehensively evaluate the overall level of PL. Building upon existing research that acknowledges and addresses the inherent tensions between PL assessment and its philosophical-conceptual foundations (such as the “journey” versus “co-existing physical literacy” perspectives) (36), and incorporating current evidence from adult PL measurement studies (37), we recommend systematically calibrating existing scales for older adults contexts and cultural dimensions. When necessary, we propose developing or adapting multidimensional assessment tools for older populations to ensure contextual validity, cultural appropriateness, and practical usability.

Third, current intervention research largely emphasizes physical training, lacking comprehensive strategic designs. Our review indicates that most interventions neglect knowledge education and motivational support, thereby limiting older adults’ intrinsic motivation for sustained physical activity engagement. Future interventions should integrate functional training with educational components and social support, forming a multidimensional approach to achieve holistic improvement.

What’s more, most of the included studies were conducted in Western cultural countries, which may limit the applicability of the findings, resulting in insufficient understanding and adaptability of PL frameworks within Eastern cultural settings. Future research should consider cross-cultural differences in physical activity perceptions and behavior patterns, developing culturally sensitive and localized interventions. For instance, integrating traditional exercises (e.g., Tai Chi) or cultural values (such as moderation and harmony) into PL frameworks may better stimulate older adults’ participation.

Moreover, greater integration of PL into nursing and public health practices is warranted. Finally, due to the mixed types of studies included in this review, it was not possible to assess the quality of the evidence or analyze the risk of bias in the sample. Currently, PL is primarily promoted by educational or athletic organizations and has not yet become routine in clinical nursing or older adults care settings. Nurses, rehabilitation therapists, and community health workers could identify older adults’ PL deficits in their routine interactions, providing simple guidance, referrals, or psychological support to effectively achieve health-promoting nursing objectives.

## 5 Conclusion

This review systematically collated the literature on physical literacy (PL) in older adults, covering its definitions, core attributes, contributing factors, health-related outcomes, and intervention practices. The results indicated that although there was no specific direct definition for older adults, their physical literacy can be identified through five core attributes: physical competence, motivation and confidence, knowledge and understanding, affective and cognitive engagement. This finding not only expands the classic framework of Whitehead/IPLA but also highlights the extended dimensions of older adults’ PL in context adaptation and social participation. Meanwhile, we note that existing evidence lacks systematic and actionable explanations regarding “adaptive adjustments” in older adults, urgently requiring further exploration of how they dynamically adjust and maintain exercise participation in real-life environments. Overall, PL represents a multidimensional and dynamic concept of health promotion. Enhancing older adults’ PL not only improves physical and mental well-being but also serves as a key factor in achieving successful aging and positive lifestyles. Future efforts should focus on developing or optimizing multi-dimensional assessment tools tailored to older adults populations based on these attributes, ensuring cultural appropriateness and practicality. This will facilitate the translation of research findings into clinical and community contexts, provide actionable references for policy-making, and advance the implementation of healthy aging strategies.

## Data Availability

The original contributions presented in the study are included in the article/Supplementary material, further inquiries can be directed to the corresponding authors.
