# Supplementary material for: Physical literacy among older adults: a scoping review of definition, attributes, contributing factors, consequences and interventions
Source: Front Public Health. 2025 Nov 5;13:1678171. doi: 10.3389/fpubh.2025.1678171 (PMC12626813; doi:10.3389/fpubh.2025.1678171)
Supplement: Supplementary file 1 [file Table_1.DOCX]

Search strategy

| Total (Searched by 2025.04.27, Items found:1828 ) | |
| --- | --- |
| 1. Pubmed (Searched by 2025.04.27, Items found: 143) | |
| #1 | "aged"[MeSH Terms] OR "geriatrics"[MeSH Terms] |
| #2 | "aged"[Title/Abstract] OR "Elderly"[Title/Abstract] OR "older*"[Title/Abstract] OR "senior*"[Title/Abstract] OR "geriatric*"[Title/Abstract] |
| #3 | #1 OR #2 |
| #4 | "physical literacy"[Title/Abstract] |
| #5 | #3 AND #4 |
| 1. Web of science (Searched by 2025.04.27, Items found: 1297) | |
| #1 | TI=(older* OR aged OR senior* OR elder* OR geriatric*) OR AB=(older* OR aged OR senior* OR elder* OR geriatric*) |
| #2 | TI=(“physical literacy”) OR AB=(“physical literacy”) |
| #3 | #1 AND #2 |
| 1. Embase (Searched by 2025.04.27, Items found: 141) | |
| #1 | 'aged'/exp |
| #2 | aged:ab,ti OR elderly:ab,ti OR ‘older*’:ab,ti OR ‘senior*’:ab,ti OR ‘geriatric*’:ab,ti |
| #3 | #1 OR #2 |
| #4 | 'physical literacy':ab,ti |
| #5 | #3 AND #4 |
| 1. CINAHL (Searched by 2025.04.27, Items found: 66) | |
| S1 | TI aged OR TI elderly OR TI older* OR TI senior* OR geriatric* |
| S2 | AB aged OR AB elderly OR AB older* OR AB senior* OR geriatric* |
| S3 | S1 OR S2 |
| S4 | TI “physical literacy” |
| S5 | AB “physical literacy” |
| S6 | S4 OR S5 |
| S7 | S3 AND S6 |
| 1. SPORTDiscus (Searched by 2025.04.27, Items found:68 ) | |
| S1 | TI ( older* OR aged OR senior* OR elder* OR geriatric* ) OR AB ( older* OR aged OR senior* OR elder* OR geriatric* ) |
| S2 | TI ( “physical literacy” ) OR AB ( “physical literacy” ) |
| S3 | S1 AND S2 |
| 1. PsycINFO (Searched by 2025.04.27, Items found: 113) | |
| S1 | tiab(older* OR aged OR senior* OR elder* OR geriatric*) |
| S2 | tiab(“physical literacy”) |
| S3 | S1 AND S2 |
